# Dataset of differentially accumulated proteins in *Mucor* strains representative of four species grown on synthetic potato dextrose agar medium and a cheese mimicking medium

**DOI:** 10.1016/j.dib.2017.02.003

**Published:** 2017-02-07

**Authors:** Stéphanie Morin-Sardin, Jean-Luc Jany, Sébastien Artigaud, Vianney Pichereau, Benoît Bernay, Emmanuel Coton, Stéphanie Madec

**Affiliations:** a Université de Brest, EA 3882 Laboratoire Universitaire de Biodiversité et d’Ecologie Microbienne, IBSAM, ESIAB, Technopôle Brest-Iroise, 29280 Plouzané, France; b Université de Brest, UMR 6539, Laboratoire des Sciences de l’Environnement Marin, LEMAR CNRS/UBO/IRD/Ifremer, Institute Universitaire Européen de la Mer, Université de Bretagne Occidentale, 29280 Plouzané, France; c Plateforme Proteogen SFR ICORE, Université de Caen Basse-Normandie, 14032 Caen Cedex, France

## Abstract

The data presented are associated with the “Proteomic analysis of the adaptative response of *Mucor* spp. to cheese environment” (Morin-Sardin et al., 2016) article [1]. *Mucor* metabolism is poorly documented in the literature and while morphology and growth behavior suggest potential adaptation to cheese for some strains, no adaptation markers to cheese environment have been identified for this genus. To establish the possible existence of metabolic functions related to cheese adaptation, we used a gel based 2-DE proteomic approach coupled to LC–MS/MS to analyze three strains from species known or proposed to have a positive or negative role in cheese production as well as a strain from a non-related cheese-species.

**Specifications Table**

**Table:** 

Subject area	Biology
More specific subject area	Cheese industry, filamentous fungi, *Mucor*, proteomics
Type of data	Tables and figures
How data was acquired	Mass spectroscopy, LC-MS/MS (AB Sciex 5800 proteomics analyzer equipped with TOF ion optics and an OptiBeam on-axis laser irradiation)
Data format	Raw, analyzed
Experimental factors	Synthetic Potato Dextrose Agar (PDA) medium and cheese mimicking medium (CA)
Experimental features	Cultures of four *Mucor* strains at 14 °C for 7 days
Data source location	Université de Brest, EA 3882 Laboratoire Universitaire de Biodiversité et d’Ecologie Microbienne, IBSAM, ESIAB, Technopôle Brest-Iroise, 29280 Plouzané, France
Data accessibility	Data are within this article

**Value of the data**

• The present dataset depicts the comparative proteome analysis of four strains representative of different *Mucor* species (“technological”, “contaminant” and non-cheese related) between two contrasting culture conditions, PDA (synthetic medium) and cheese agar (cheese mimicking medium).
• Differential morphologies and growth capacities among species and media were confirmed and a total of 289 proteins differentially accumulated were identified.
• Data reported here deepened our understanding on *Mucor* species metabolism and strongly suggested that adaptative response is species-specific.

## 1 Data

**Fig. 1:**
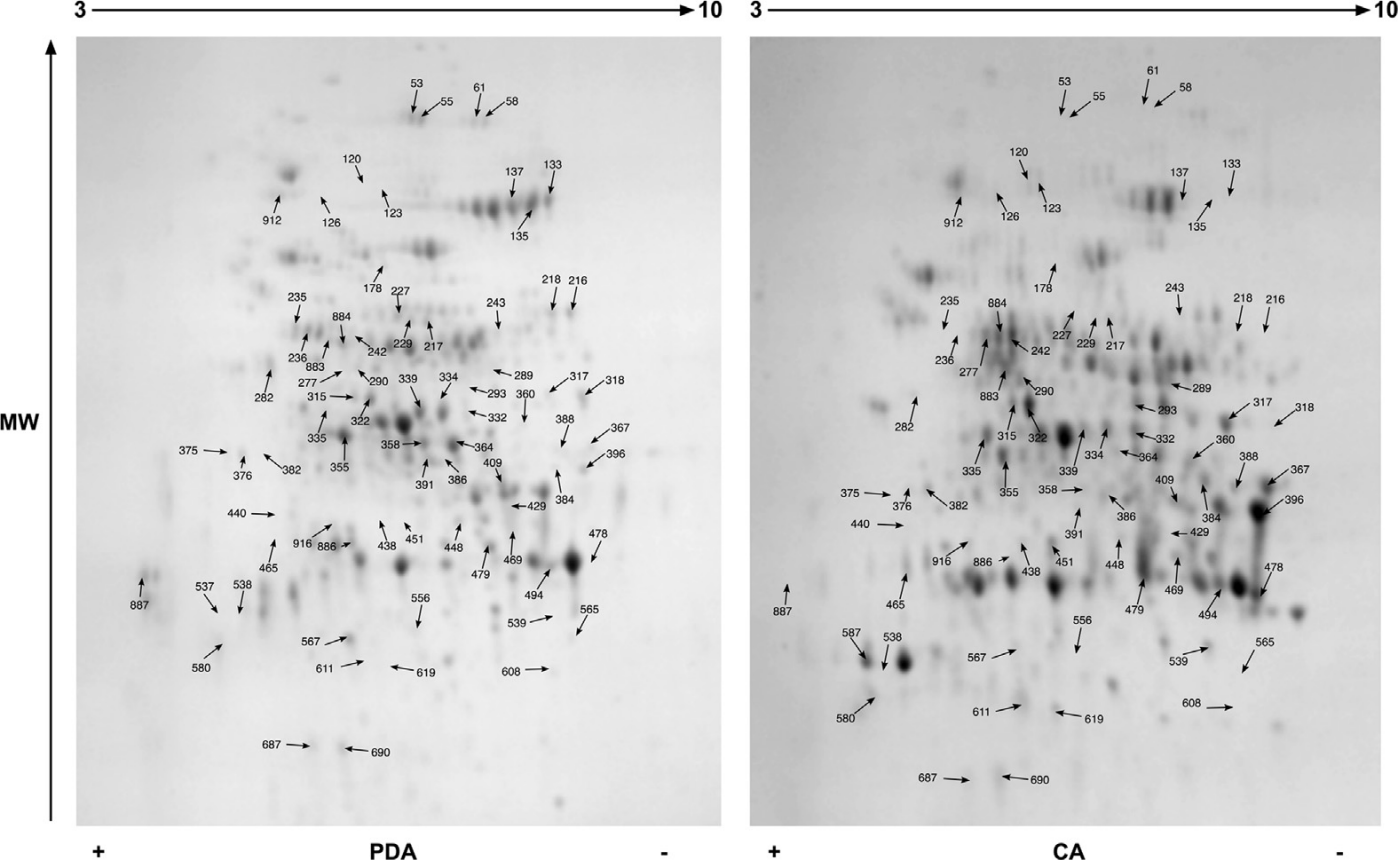
Consensus bi-dimensional protein gels (pH 3–10, SDS-PAGE 12%) obtained for *M. racemosus* UBOCC-A-109155 on potato dextrose agar (PDA) and cheese agar (CA) media. Arrows correspond to protein spots differentially expressed (fold ≥1.6; *p* value ≤0.02) and excised for mass spectrometry analysis. A minimum of 6 replicates were done for each strain and medium.

**Fig. 2:**
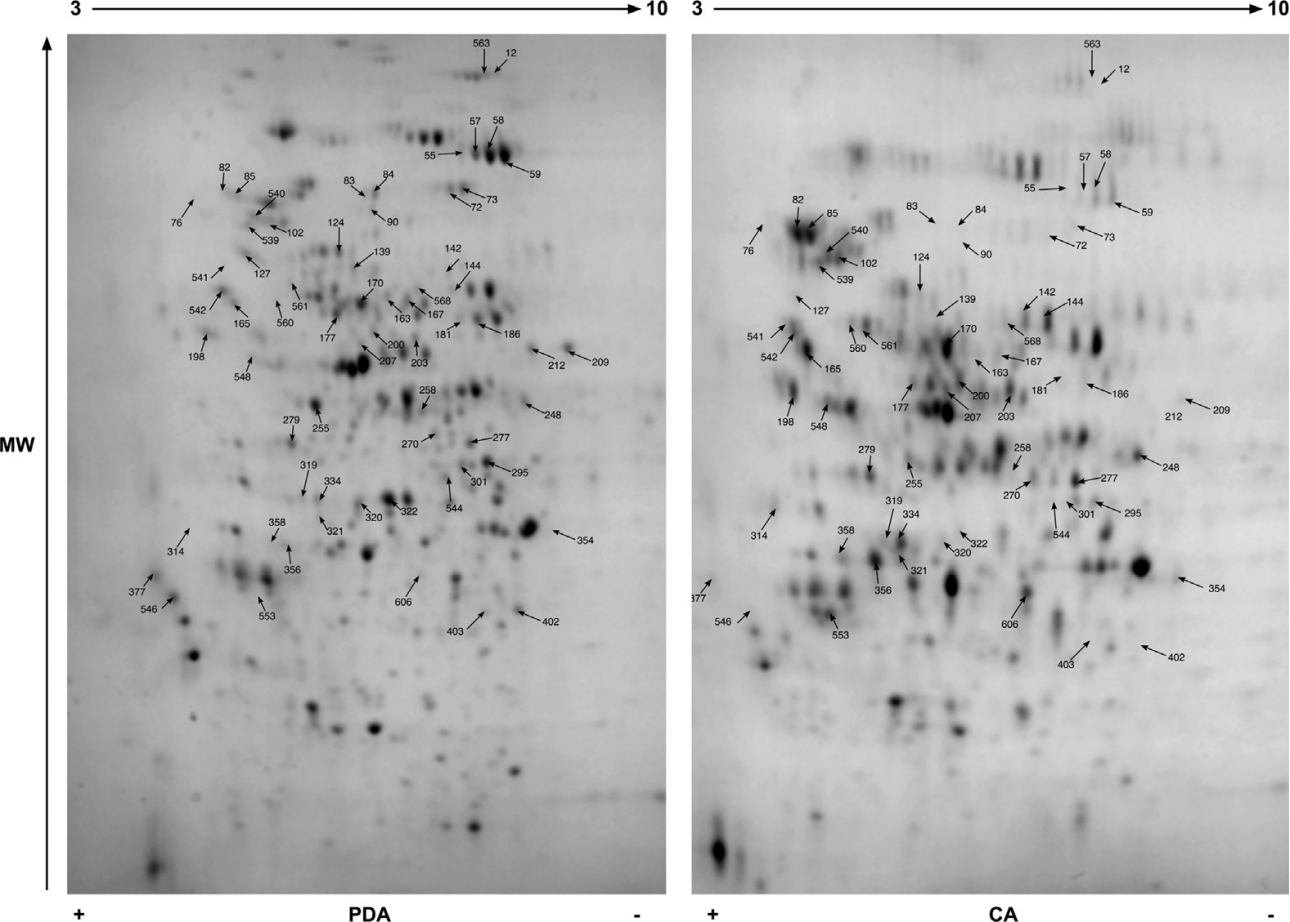
Consensus bi-dimensional protein gels (pH 3–10, SDS-PAGE 12%) obtained for *M. lanceolatus* UBOCC-A-109153 on potato dextrose agar (PDA) and cheese agar (CA) media. Arrows correspond to protein spots differentially expressed (fold ≥1.6; *p* value ≤0.02) and excised for mass spectrometry analysis. A minimum of 6 replicates were done for each strain and medium.

**Fig. 3:**
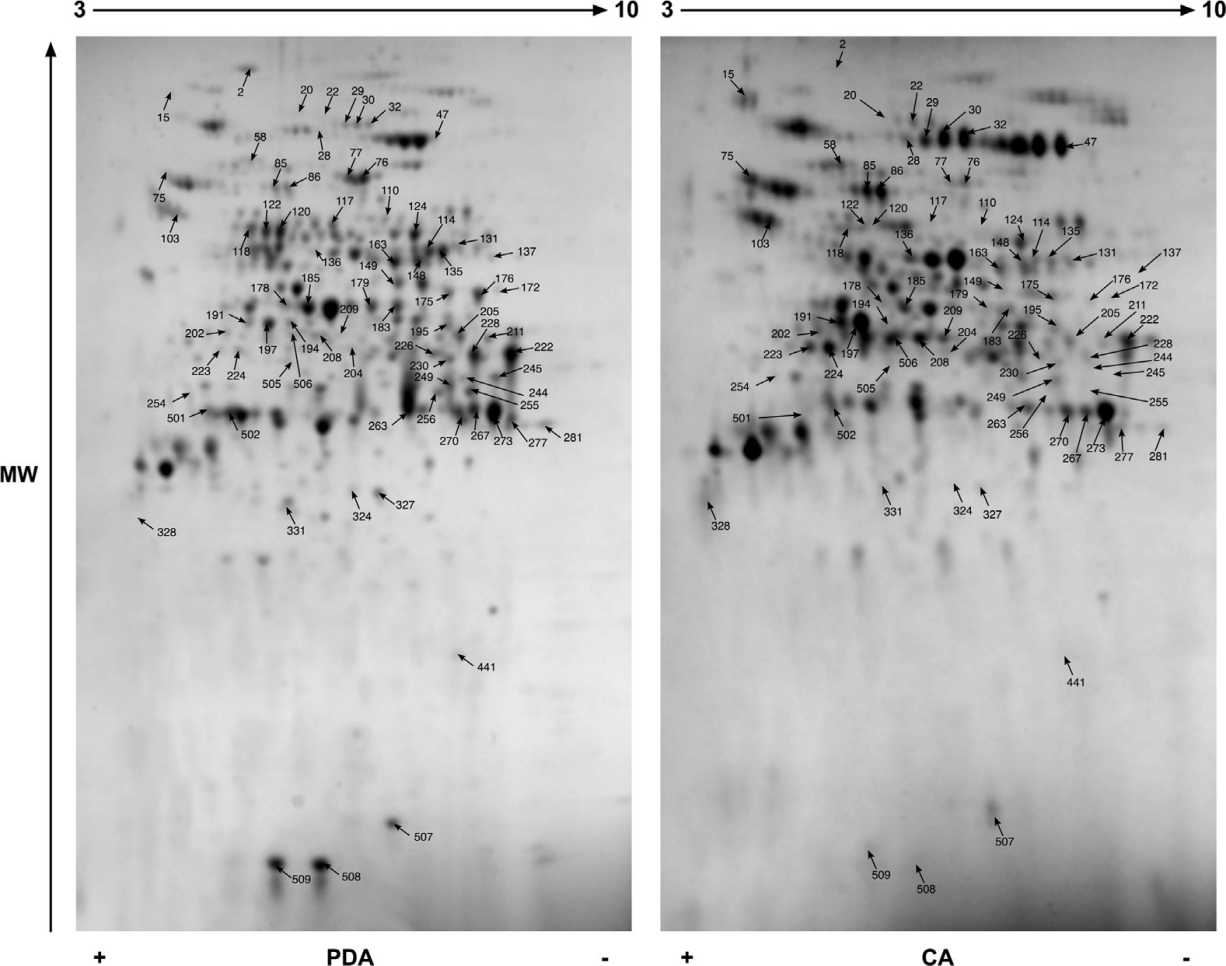
Consensus bi-dimensional protein gels (pH 3–10, SDS-PAGE 12%) obtained for *M. circinelloides* CBS 277-49 on potato dextrose agar (PDA) and cheese agar (CA) media. Arrows correspond to protein spots differentially expressed (fold ≥1.6; *p* value ≤0.02) and excised for mass spectrometry analysis. A minimum of 6 replicates were done for each strain and medium.

**Fig. 4:**
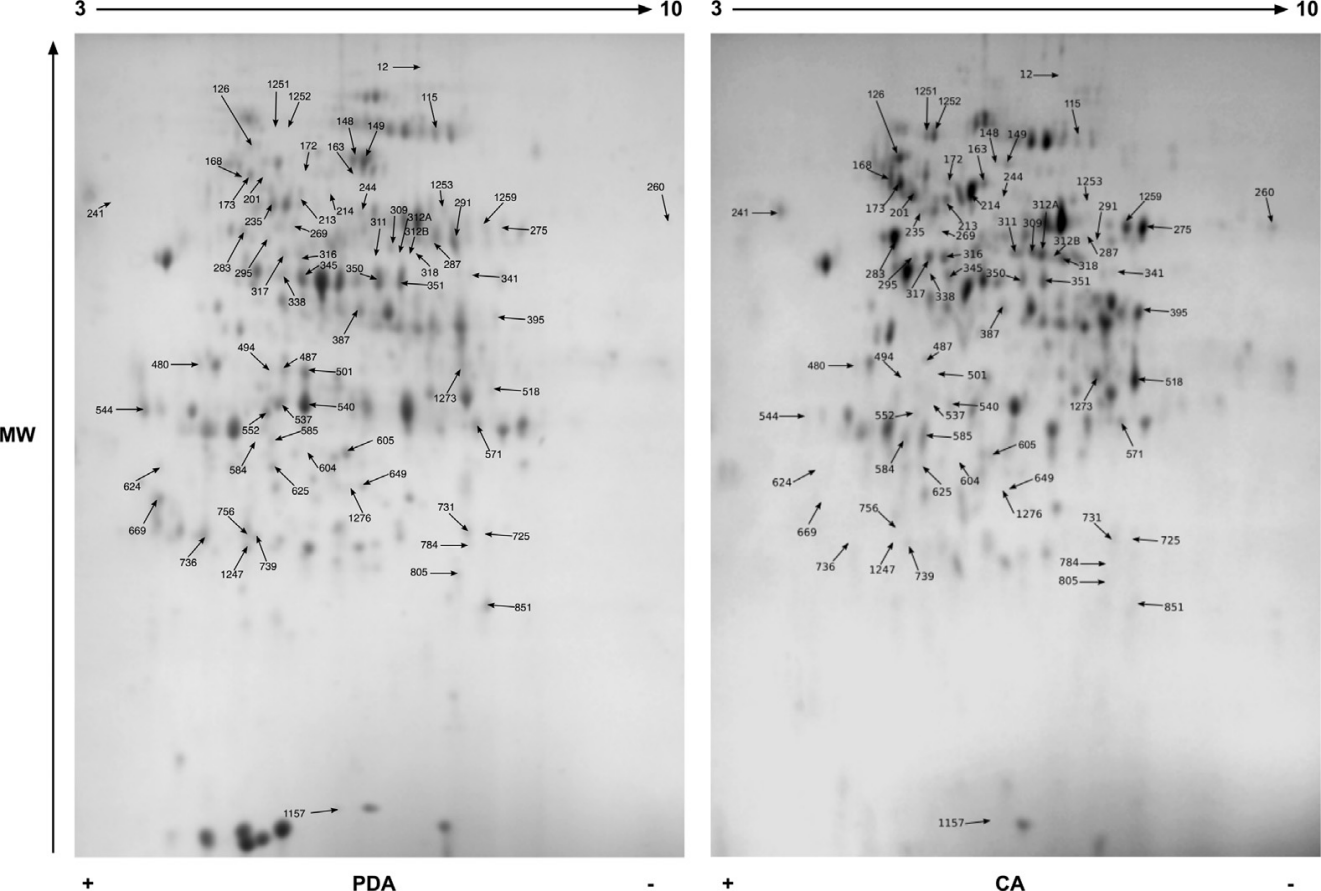
Consensus bi-dimensional protein gels (pH 3–10, SDS-PAGE 12%) obtained for *M. endophyticus* CBS 385-95 on potato dextrose agar (PDA) and cheese agar (CA) media. Arrows correspond to protein spots differentially expressed (fold ≥1.6; *p* value ≤0.02) and excised for mass spectrometry analysis. A minimum of 6 replicates were done for each strain and medium.

**Fig. 5:**
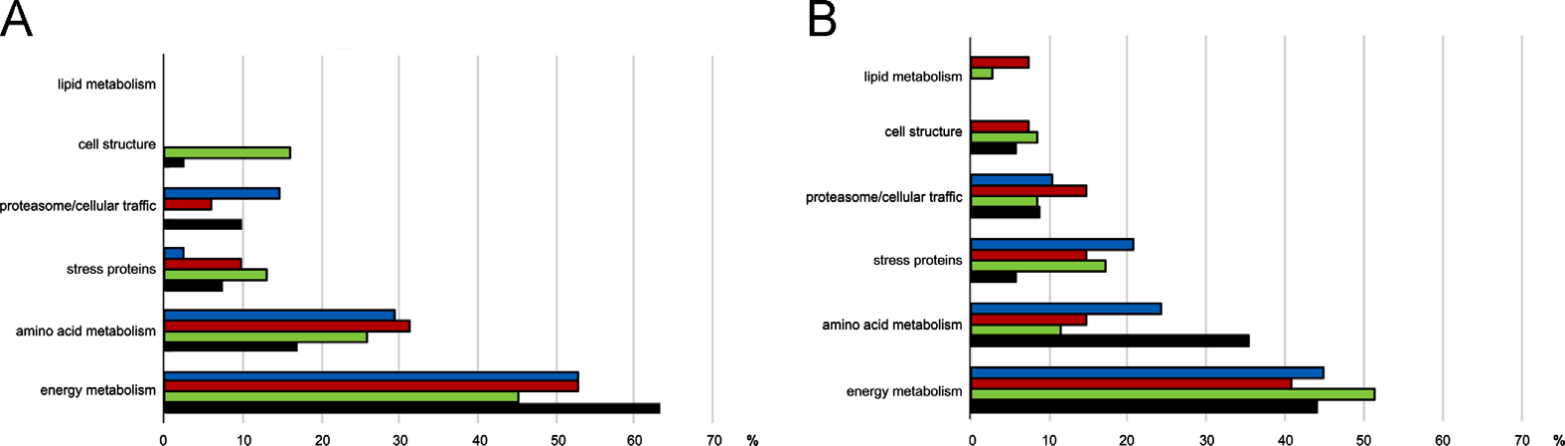
Functional repartition of the over-accumulated proteins identified on potato dextrose agar (PDA) (A) and cheese agar (CA) (B) media for *M. racemosus* UBOCC-A- 109155 (black), *M. lanceolatus* UBOCC-A-109153 (green), *M. circinelloides* CBS 277-49 (red) and *M. endophyticus* CBS 385-95 (blue). Proportion (%) of over-accumulated proteins is given per family. Each protein was assigned to a single functional category that was considered as the best assignment according to data mining using Blast2Go.

Data reported here describe the comparison of the bi-dimensional protein gels obtained for *M. lanceolatus* UBOCC-A-109153, *Mucor racemosus* UBOCC-A-109155, *Mucor circinelloides* CBS 277-49 and *Mucor endophyticus* CBS 385-95 on PDA and CA media (Fig. 1, Fig. 2, Fig. 3, Fig. 4), the identification of the proteins over-accumulated on each media for the 4 species including results of homologies search with *Saccharomyces cerevisiae* proteome (Supplementary Tables S1–S8) and the functional repartition of these identified proteins using GO analysis (Fig. 5).

## 2 Experimental design, materials and methods

### 2.1 Fungal cultures

Four *Mucor* strains: *M. lanceolatus* UBOCC-A-109153, *M. circinelloides* CBS 277-49, *M. racemosus* UBOCC-A-109155 provided by the Université de Bretagne Occidentale Culture Collection (UBOCC, France, http://www-tmp.univ-brest.fr/souchotheque) and *M. endophyticus* CBS 385-95 originating from the Centraal Bureau Voor Schimmelculture (CBS, The Netherlands, http://www.cbs.knaw.nl/) were selected according to their known or proposed role in cheese production [1]. The four strains were grown on potato dextrose agar (PDA) medium (DIFCO Laboratories, USA) [2] in the dark at 25 °C. Spore suspensions of each strain were produced as previously described [3] and adjusted at 10^7^–10^8^ spores mL^−1^ prior to storage at −80 °C before use. These spore suspensions were diluted into Potato Dextrose Broth solution (DIFCO Laboratories, USA) to obtain a 10^6^ spores mL^−1^ final concentration. Ten microliters of the diluted spore suspension were used for inoculation of plates of either PDA medium or Cheese Agar (CA, a cheese mimicking medium) [4], [5], covered by a sterile cellophane sheet. Petri plates were then stored in airtight boxes and incubated 7 days at 14 °C.

### 2.2 Protein extraction

After incubation, each mycelium was ground using a mortar and a pestle in the presence of liquid nitrogen. One hundred milligrams of the obtained powder were homogenized 2 h at 4 °C in 10 ml of 75 mM Tris–HCl (pH 7) containing 1 tablet of complete EDTA free protease inhibitor cocktail (Roche Diagnostics GmbH, Mannheim, Germany) and 0.07% DTT (GE Healthcare, Saclay, France). After centrifugation (9000 rpm, 10 min, 4 °C), the supernatants containing the solubilized proteins were enzymatically treated by a nuclease mix (GE Healthcare) to remove nucleic acids. Samples were then precipitated at 4 °C using a mixture of TCA/acetone with 20% trichloroacetic acid and 0.14% DTT (1/1:v/v, overnight). After centrifugation (9000 rpm, 10 min, 4 °C), pellets were washed 4 times by adding 400 μl of a solution of Tris–HCl pH 8.8 (50 mM)/80% acetone containing traces of bromophenol blue, used as a pH indicator. Each washing step was followed by centrifugation (9000 rpm, 10 min, 4 °C). The recovered pellets were dried at room temperature and resuspended in 250 μl of Destreak rehydration solution (GE Healthcare, Saclay, France) before being placed at 4 °C during 6 h then transferred to Eppendorf at −80 °C prior to analysis. Protein concentrations were determined using a modified Bradford assay [6] and all samples were adjusted to 1000 μg of proteins in 250 μl of Destreak containing 1% IPG buffer (pH 3–10) (GE Healthcare, Saclay, France).

### 2.3 Two-dimensional electrophoresis

Prior to isoelectric focusing, IPG strips (pH 3–10, 13 cm, GE Healthcare, Saclay, France) were passively rehydrated with 250 μl of protein solution for 16 h. Isoelectric focusing was conducted as follows: 250 V for 15 min, 500 V for 2 h, 1000 V for 1 h, gradual voltage increase to 8000 V for 2 h 30, then 8000 V for 3 h, and final reduction to 500 V for a maximum of 20 h, all carried out at 20 °C (Ettan IPGphor 3, GE Healthcare, Saclay, France). To prepare the second dimension, corresponding to a SDS-PAGE, strips were incubated in equilibration buffer (50 mM Tris–HCl pH 8.8, 6 M urea, 30% glycerol, 2% SDS and 0.002% bromophenol blue) for two 15 min-periods, first with 1 g l^−1^ of DTT and then with 48 g l^−1^ of iodoacetamide [7]. IPG strips were then placed on top of a lab-cast 15 cm×15 cm SDS-PAGE gel containing 12% polyacrylamide, which were run in 10 °C thermo-regulated device (Multitemp IV thermostatic circulator, GE Healthcare, Saclay, France) at 10 mA per gel for 1 h and then 30 mA per gel until complete migration [8]. Gels were subsequently stained with Coomassie Blue (PhastGel, GE Healthcare, Saclay, France) overnight and unspecific coloration was destained by successive baths in a H_2_O/methanol/acetic acid: 70/30/7 solution.

### 2.4 Gel image analysis

A minimum of 6 gels per condition were analyzed. 16-bit tif images of the gels obtained using G:BOX (SynGene) were aligned and spots were detected and quantified using the Progenesis SameSpots software (Nonlinear dynamics, v.4.5, Newcastle upon Tyne, UK) using the automated algorithm. ANOVA statistical analyses using the statistics module of SameSpot were done for spot selection by comparing abundance on PDA versus CA. Significantly differentially expressed proteins at *P*≤0.02 with an absolute fold change ≥1.6 were targeted.

### 2.5 Mass spectrometry

Protein spots for which abundance differed between media were excised from gels and prepared for MS analysis. Excised spots were washed with milliQ water and destained in 50 mM ammonium bicarbonate (BICAM 100 mM/acetonitrile (ACN) (1:1)), and then dehydrated in 100% ACN. Gel pieces were vacuum-dried rehydrated with BICAM containing 0.25 μg sequencing grade porcine trypsin (Promega, Madison, USA) and incubated 20 h at 37 °C. Peptides were extracted from the gels by alternative washing with 50 mM BICAM and ACN, and with 5% formic acid and ACN as previously described [7]. Between each step, the supernatants were pooled (30 μL) and finally concentrated by evaporation using a centrifugal evaporator (Concentrator 5301, Eppendorf, Hamburg, Germany).

MS experiments were carried out on an AB Sciex 5800 proteomics analyzer equipped with TOF ion optics and an OptiBeam on-axis laser irradiation with 1000 Hz repetition rate. The system was calibrated immediately before analysis with a mixture of Angiotensin I, Angiotensin II, Neurotensin, ACTH clip (1-17), ACTH clip (18-39) and mass precision was better than 50 ppm. After tryptic digestion, the dry sample was resuspended in 10 μL of 0.1% trifluoroacetic acid (TFA). A 1 μl volume of this peptide solution was mixed with 10 μL of alpha-cyano-4-hydroxy- cinnamic-acid (CHCA) matrix prepared in a diluent solution of 50% ACN with 0.1% TFA. The mixture was spotted on a stainless steel Opti-TOF 384 targets; the droplet was allowed to evaporate before introducing the target into the mass spectrometer. All acquisitions were taken in automatic mode. A laser intensity of 3400 was typically employed for ionizing. MS spectra were acquired in the positive reflector mode by summarizing 1000 single spectra (5×200) in the mass range from 700 to 4000 Da. MS/MS spectra were acquired in the positive MS/MS reflector mode by summarizing a maximum of 2500 single spectra (10×250) with a laser intensity of 3900. For the tandem MS experiments, the acceleration voltage applied was 1 kV and air was used as the collision gas. Gas pressure medium was selected as settings. The fragmentation pattern was used to determine the sequence of the peptide.

### 2.6 Protein identification

Mass spectrometry data were analyzed using the Mascot 2.4.0 program (Matrix Science Ltd.). The variable modifications allowed were as follows: C-Carbamidomethyl, K-acetylation, methionine oxidation, and dioxidation. “Trypsin” was selected as enzyme, and 3 miscleavages were also allowed. Mass accuracy was set to 300 ppm and 0.6 Da for MS and MS/MS mode respectively. A public database (*M. circinelloides* assembly v2*,* 97356 entries) or unpublished genome data available in our laboratory (*M. lanceolatus,* 17940 entries; *M. racemosus*, 3380 entries) were used. Protein identification was considered as unambiguous when a minimum of two peptides matched. All protein sequences were subjected to homology searches using the Blast algorithm available on NCBI (www.ncbi.nlm.nih.gov) and Uniprot (http://www.uniprot.org), with default parameters. Assignment of information about the biological function of over-accumulated proteins identified was done using Blast2Go (https://www.blast2go.com*, version* 3.2.7). A network analysis was then performed using the known and predicted protein interaction STRING database (www.string-db.org, *version* 10.0) which includes both direct (physical) and indirect (functional) associations [1]. Because none *Mucor* or other early diverging fungi data were present in this database, the analysis was carried out using the interactions known in *Saccharomyces cerevisiae.*
